# Chrononutrition in the Prevention and Management of Metabolic Disorders: A Literature Review

**DOI:** 10.3390/nu16050722

**Published:** 2024-03-01

**Authors:** Maria Mentzelou, Sousana K. Papadopoulou, Evmorfia Psara, Gavriela Voulgaridou, Eleni Pavlidou, Odysseas Androutsos, Constantinos Giaginis

**Affiliations:** 1 Department of Food Science and Nutrition, School of Environment, University of the Aegean, 81400 Lemnos, Greece; maria.mentzelou@hotmail.com (M.M.); fnsd21013@fns.aegean.gr (E.P.); elen.p.pavl@gmail.com (E.P.); 2 Department of Nutritional Sciences and Dietetics, School of Health Sciences, International Hellenic University, 57400 Thessaloniki, Greece; souzpapa@gmail.com (S.K.P.); gabivoulg@gmail.com (G.V.); 3 Laboratory of Clinical Nutrition and Dietetics, Department of Nutrition and Dietetics, School of Physical Education, Sport Science and Dietetics, University of Thessaly, 42132 Trikala, Greece; oandroutsos@uth.gr

**Keywords:** chrononutrition, metabolic diseases, time-restricted eating, time-restricted feeding, circadian rhythms

## Abstract

Background: The concept of time-restricted eating (TRE) or time-restricted feeding (TRF) promotes daily periods of feeding and fasting to determine whole-body physiology. Chronic misalignment of circadian rhythms or chrono-disruption is related to an increased risk of diverse metabolic disorders. The progression of non-communicable diseases seems to be affected by the timing of meals. As a result, intermittent fasting is a promising approach for their management. The aim of the present literature review is to examine and scrutinize the TRE protocols in the fields of prevention and management of metabolic disorders. Methods: This is a thorough literature review of the reported associations among circadian rhythm, metabolic disorders, diabetes mellitus, obesity, TRE, TRF, dietary habits, circadian disruption, cardiovascular diseases, atherosclerosis, and non-alcoholic fatty liver to find the already existing clinical studies from the last decade (2014–2024) in the most precise scientific online databases, using relevant specific keywords. Several inclusion and exclusion criteria were applied to scrutinize only longitudinal, cross-sectional, descriptive, and prospective clinical human studies. Results: The currently available clinical findings remain scarce and suggest that chrononutrition behaviors such as TRE or TRF may promote several metabolic benefits, mainly in body weight control and fat loss. Improvements in glucose levels and lipid profiles are currently quite controversial since some clinical studies show little or no effect. As far as liver diseases are concerned, the efficacy of intermittent fasting seems to be stronger in the management of non-alcoholic fatty liver disease due to body weight decline and fat loss. Conclusions: Even if there has been a gradual increase in clinical studies in the last few years, providing promising perspectives, currently, there is no conclusive evidence for the role of chrononutrition in metabolic disorders. Future studies should be well-designed with longer duration and larger sample sizes. Moreover, it is important to examine the best timing of the eating window and its feasibility.

## 1. Introduction

Chrononutrition is a field of research in nutritional sciences that investigates the health impact of three different dimensions of feeding behavior: regularity of meals, frequency, and timing of food intake [1]. These are distinguished into early chronotypes and late chronotypes. The evening type has been associated with unhealthy food choices, night binge eating, and multiple metabolic disorders, whereas morning individuals are associated with better mental health [2]. In humans, all the physiological functions are adapted to the light/dark cycles [2]. The periodicity of this clock is approximately 24 h, and it is therefore called a circadian rhythm. The suprachiasmatic nucleus (SCN) of the hypothalamus functions as a “central clock” and coordinates “peripheral clocks” through diverse hormonal and neuronal signals, sustaining the rhythmic gene expression of oscillating genes [3]. At the heart of the molecular complex are the core transcription factors circadian locomotor output cycles kaput (CLOCK) and brain and muscle Arnt-like protein-1 (BMAL1) [4]. CLOCK and BMAL1 drive the transcription of their own repressors, period (PER) and cryptochrome (CRY), leading to a strongly self-regulated feedback loop. Circadian rhythm alterations could have metabolic consequences in subjects carrying specific single nucleotide polymorphisms in other genes related to the circadian clock, such as *PNPLA3*, *PPARY*, *STAT3*, and *PPARGCα* [4].

In the last few decades, it has been widely recognized that nutritional components, including macro- and micro-nutrients and naturally occurring bioactive compounds, can (directly or indirectly) regulate central and peripheral clocks. For example, high-fat diets and the daily allocation of macronutrients appear to act like disruptors, exerting detrimental effects on several metabolic health factors [2,5]. Moreover, it is important to orient the meal timing, the frequency, and the patterns of energy intake within the day/night cycle [2]. During the day, genes related to glycogenesis and lipogenesis are active, while during the night, genes related to growth, repair, glycogenolysis, and lipolysis are triggered [6]. Remarkably, any trouble in these time ranges could be associated with impairment of energy metabolism [3,7].

Currently, it is well established that the global prevalence of diabetes, obesity, atherosclerosis, and non-alcoholic fatty liver disease has become a major public health problem and a burden on healthcare systems [8,9,10]. Their epidemic proportions urgently require new and effective approaches for their management worldwide. In this aspect, chronic misalignment of circadian rhythms or chrono-disruption has been related to an increased risk of metabolic disorders due to extended exposure to artificial light, increased shift work, sedentarism, untimely and frequent snacking, and jetlag, as well as industrialization [11,12]. Currently, and in view of the above considerations, there is no simple solution to address the escalating risk of these diseases.

**Figure 1:**
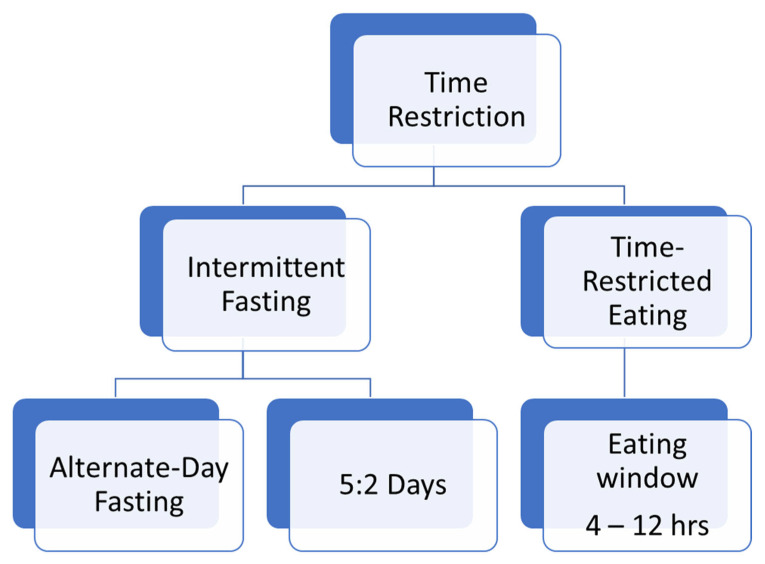
Types of time-restriction diet protocols.

The concept of time-restricted eating (TRE) or time-restricted feeding (TRF) promotes daily periods of feeding and fasting to determine whole-body physiology [13]. TRE, a subtype of intermittent fasting (IF) regimen that calls for a set window of time for eating and fasting within each 24 h period, has attracted the research interest as a successful approach for the prevention and co-treatment of obesity and metabolic disorders [14]. The window of eating ranges between 4 and 12 h, without intentional changes in caloric intake [15,16]. An example of such a modification is alternate-day fasting (ADF), which involves eating one day and then fasting the next day. On the day of fasting, one meal (most often consumed at lunch) is usually eaten, which does not exceed 25% of the daily caloric requirement. Another example of IF modification is the introduction of 1 or 2 days of fasting per week (5:2 days). The fasting day includes either the complete elimination of food or the reduction in calories to a minimum level [15,16]. However, due to the limited feeding time, a mild caloric deficit is usual. On the other hand, TRF is more than just a caloric restriction; it also synchronizes the feeding time with the awake/active phase when the body is best able to metabolize food [2,5,6]. The most common types of the above time-restriction diet protocols are depicted in Figure 1.

The progression of non-communicable diseases appears to be affected by the timing of meals. As a result, IF seems a promising approach for their effective management. It would be worthwhile to mention that in the application of TRE protocols, the implementation of current guidelines for diet composition and quality of diet, including the intakes of whole grain, plant-based diets, limiting ultra-processed food, and portion control, is highly crucial. Chrononutrition behaviors such as TRE or TRF may considerably positively improve metabolic disorders. However, the findings from the international scientific bibliography data are still quite scarce and remain conflicting and inconclusive [17]. In this aspect, the aim of the present review is to examine and scrutinize the initial research interest in TRE as a healthy nutritional intervention among humans that could contribute to the regulation of circadian rhythm, improving metabolism, and overall metabolic health in the fields of prevention and management of metabolic disorders.

## 2. Methods

This is an in-depth, comprehensive literature review of the clinical evidence from the last decade (2014–2024) concerning the role of chrononutrition and circadian rhythms in the prevention and management of metabolic disorders. In fact, the most accurate scientific databases, e.g., PubMed, Scopus, and Web of Science, were comprehensively searched utilizing relevant keywords, such as “circadian rhythm”, “metabolic disorders”, “diabetes mellitus”, “obesity”, “time restricted eating”, “time restricted feeding”, “dietary habits”, “circadian disruption”, “cardiovascular diseases”, “atherosclerosis”, “non-alcoholic fatty liver”, and “thyroid diseases”, to retrieve the clinical studies from the last ten years (2014–2024). The results were filtered based on relevance, and the most relevant ones were selected and quoted below. Only clinical human studies with a strong protocol design and written in the English language were included in this review. Review articles, case reports, and abstracts in conference proceedings were excluded from the final analysis.

**Figure 2:**
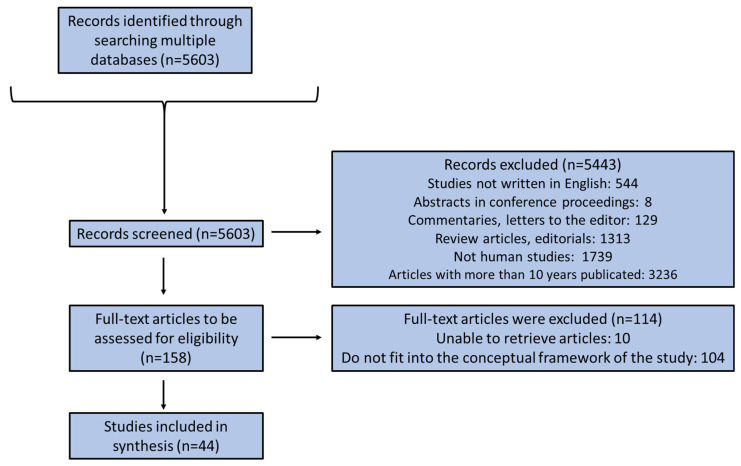
Flow chart diagram of study enrollment.

The search was supplemented by scanning the reference lists of relevant reviews, hand-searching key journals, and reviewing commentaries and editorials. The retrieved surveys were, additionally, comprehensively checked for related studies quoted in their text. All authors acted as reviewers. To enhance the consistency among reviewers, all of them screened the retrieved publications, discussed the results, and amended the screening and data extraction manual before initiating screening for this review. The reviewers working in pairs sequentially evaluated the titles, abstracts, and then the full text of all publications identified by our searches for potentially relevant publications. We resolved disagreements on study selection and data extraction by consensus and discussion with all the authors/reviewers if required. The findings were selected based on relevance, and the most relevant ones were chosen and mentioned below according to the flow chart diagram depicted in Figure 2.

## 3. Results

### 3.1. TRE or TRF and Obesity

There are several clinical studies that assessed the effects of TRE or TRF on obesity. The currently existing studies are summarized in Table 1. This table includes nine clinical studies with adequate, well-organized methodologies.

Catenacci et al. compared the effects of ADF, an IF approach in which you can fast on one day and then you can eat what you want the next day, on changes in body weight and insulin sensitivity index with changes in a standard weight-loss diet with moderate daily caloric restriction [18]. The result of ADF intervention was only a 1.1 Kg greater decline, possibly due to under-reported food intake and a decrease in non-resting energy expenditure in the ADF group. Thus, future studies are needed to explore these mechanisms [18]. Moreover, Chair et al. examined the effects of ADF and 16/8 time-restricted fasting (16/8 TRF) on weight loss, blood glucose, and lipid profile in overweight and obese adults with prediabetes [19]. The above intervention lasted 3 weeks. The findings of this study showed improvements in both body weight and body mass index (BMI) [19]. Moreover, in a randomized controlled trial (RCT), Cai et al. evaluated the effects of ADF/TRF on the body weight and lipid profile of individuals with non-alcoholic fatty liver disease (NAFLD) [20]. Anthropometric measurements were collected, and plasma lipids were analyzed enzymatically. ADF/TRF seemed to be an effective intervention for individuals with NAFLD that can achieve body weight loss. In addition, this study found improvements in total cholesterol in the ADF group. It is important to note that TRF decreased the BMI, contributing to the deterioration of NAFLD progression [20].

Furthermore, Wilkinson et al. investigated the efficacy of TRE in combination with pharmacotherapy in individuals diagnosed with metabolic syndrome [21]. It was shown that ten-hour TRE reduced human body weight. These benefits exerted an additive effect on the used medications. However, this study had some limitations as it was designed as an unblinded, single-arm study. Moreover, it had several differences in the use of pharmacotherapy, and it included a small sample size [21]. In another study, Gabel et al. examined the effects of 8 h TRF on the gut microbiome in adults affected by obesity [22]. The findings of this study showed that body weight decreased by 2 ± 1 kg. Nevertheless, this study had certain limitations. In fact, it had a no-intervention control group and included a small sample size [22]. In addition, Schübel et al. conducted an RCT to examine whether intermittent calorie restriction (ICR) could exert stronger effects on adipose tissue gene expression, anthropometric and body composition measures, and circulating metabolic biomarkers than continuous calorie restriction (CCR) and a control regimen [23]. Log relative weight change over the intervention phase was −7.1 ± 0.7% with ICR, −5.2 ± 0.6% with CCR, and −3.3 ± 0.6% with the control group. Moreover, this study provided evidence for a slight body weight regain after initial weight loss in the ICR group, which was not observed in the CCR group [23].

Sundfor et al. examined the one-year effects of intermittent versus continuous energy restriction on body weight loss [24]. Both diets resulted in an equivalent reduction in energy intake. Regardless of the type of diet chosen, weight loss was similar among participants in the intermittent and continuous energy restriction groups. Concerning body weight maintenance after six months of intervention, the energy restriction was not achieved by most individuals [24]. The main strength of this study is the generalizability of its results due to the high retention rates in both groups and the inclusion of similar proportions of men and women. Further long-term studies lasting for at least two years or more may help determine whether intermittent energy restriction could be more effective compared with continuous energy restriction [24]. In another RCT, Lowe et al. assessed the effect of TRE on body weight in individuals with increased BMI [25]. This study found a significant decrease in body weight in the TRE group and a non-significant decrease in body weight in the CMT group. However, it should be noted that the main limitations of this study were the self-reported measures and the lack of measurement of some crucial parameters related to protein intake [25]. Lastly, Schroder et al. aimed to explore the impact of TRF on body composition and the association of body weight loss with metabolic and cardiovascular risks in obese middle-aged women [26]. The adopted TRF protocol was 16 h without any energy intake followed by 8 h of normal food intake. The findings of this study revealed that TRF may be considered an effective approach to decrease body weight. However, the main limitations of this study were the lack of randomization in the clinical trial and the absence of specific inclusion criteria concerning the detailed nutritional aspects of the assigned individuals [26].

### 3.2. TRE or TRF and Diabetes Mellitus

There are several clinical studies that assessed the effect of TRE or TRF on diabetes mellitus. The currently existing studies are summarized in Table 2. This table includes 10 clinical studies with adequate, well-organized methodologies.

Che et al. designed an RCT that explored the potential effects of TRF in overweight patients diagnosed with type 2 diabetes [27]. The findings of this study demonstrated that the TRF group had significant improvements in hemoglobin A1c (HbA1c) over 12 weeks. The main limitations of this study were the short time of intervention and the self-reported data, which may include recall bias [27]. In addition, Pavlou et al. conducted a 6-month RCT comparing the effects of an 8 h TRE group (eating ad libitum between 12:00 pm and 8:00 pm), a CR group (25% caloric restriction daily) and a control group (instructed to maintain their body weight and daily eating habits) [28]. Participants in the TRE and CR groups did not differ concerning glycemic control. However, the basic limitations of this study were the short duration of the clinical trial and the lack of blinding of participants [28]. Another clinical study investigated the potential effects of TRE in individuals diagnosed with metabolic syndrome [28]. This study demonstrated that a 10 h TRE intervention over 12 weeks significantly reduced the levels of HbA1c. However, this study had a severe limitation concerning its design as it was unblinded and included a small sample size [21].

Furthermore, another clinical study examined the potential effects of ADF and 16/8 TRF on body weight, blood glucose, BMI, waist circumference (an index of abdominal obesity), and lipid profile in overweight and obese individuals with prediabetes [19]. The above study showed that both the ADF and 16/8 TRF groups achieved significantly favorable results in terms of reducing body weight, BMI, and waist circumference across the study period compared to the control group. The main limitations of this study were the short follow-up period and the small sample size. Hence, more studies are needed using a more accurate methodology and longer duration, which are highly recommended to verify the results and to better understand the underlying mechanisms [19]. Carter et al. examined the effects of a 2-day intermittent energy restriction diet compared with continuous energy restriction in individuals with type 2 diabetes at a 24-month follow-up and 12 months after the completed intervention [29]. This study indicated that HbA1c levels did not differ between the two groups [29]. Gambel et al. compared the effects of ADF to daily CR on body weight and glucoregulatory factors in patients with insulin resistance and overweight/obesity [30]. This study provided evidence for the positive effects of IF versus daily CR on glucoregulatory factors. The strengths of this study were the inclusion of a no-intervention control group and the trial duration. However, this study also had some limitations concerning the use of homeostatic model assessment for insulin resistance (HOMA-IR) to assess insulin resistance and the lack of assessment of the number of prediabetic individuals in the study sample [30]. In this aspect, future studies are highly recommended to confirm the potential beneficial effects of IF in individuals with prediabetes who implement hyperinsulinemic-euglycemic clamps to assess insulin resistance and sensitivity [30].

In addition, Kunduraci et al. aimed to examine the effects of TRE on improving glycemic biomarkers among adults with metabolic syndrome [31]. The intervention and control groups did not differ significantly in glycemic measures [31]. The main limitations of this study were that the randomization was stratified only by sex and age, as well as the sample size, which was quite small. Thus, larger RCTs should further explore the clinical effectiveness of fasting programs in metabolic syndrome patients [31]. In another study, Obermayer et al. assessed the efficacy of IF in individuals with type 2 diabetes [32]. This study found that after 12 weeks of intervention, the IF group exhibited a significant reduction in total daily insulin dose of 9 ± 10 IU as opposed to the control group, with an increase of 4 ± 10 IU. The major strength of this study was its randomized controlled design in individuals with type 2 diabetes and the monitoring of the resting metabolic rate (RMR) and physical activity levels [32]. A limitation of the study was the unblinded use of a glucose monitoring system (CGM) for the study participants, which may have influenced participants’ dietary habits [32]. Peeke et al. also investigated the effectiveness of a daily 14 h metabolic fast combined with a commercial body weight management program on body weight and fasting blood glucose (FBG) in individuals with obesity [33]. Participants in both groups demonstrated a reduction from baseline in fasting blood glucose. However, a basic limitation of this study was its virtual design. Moreover, important drawbacks of this study were the small sample size and its short duration. Thus, it was suggested that longer and larger studies are highly required to verify the differences between the group focused on body weight and the group evaluating blood glucose [33]. Lastly, Khalfallah et al. examined the efficacy of both IF and low-carb diet (LCD) on microvascular and macrovascular outcomes in prediabetic patients [34]. This study demonstrated a significantly greater reduction in body weight, BMI, waist circumference, and fat percentage in group I (IF–LCD); however, no significant difference in visceral fat or skeletal muscle percentage between the two groups was noted [34].

### 3.3. TRE or TRF and Cardiovascular Diseases

Some clinical studies have assessed the effect of TRE on cardiovascular diseases. The currently existing studies are summarized in Table 3. This table includes seven clinical studies with adequate, well-organized methodologies.

Cienfuegos et al. compared the effect of two popular forms of TRF (4 h and 6 h) on body weight and cardiometabolic risk factors in adults with obesity versus a control group that had no meal timing restrictions [35]. Reductions in insulin resistance and oxidative stress were noted, highlighting these regimens in preventing cardiometabolic disease. However, this study had some limitations concerning the small size of the sample, the indicators of oxidative stress, and the lack of cross-over design [35]. In addition, Sutton et al. determined whether early TRF could reduce cardiovascular health markers and the independent health effects of IF [36]. Early TRF was found to improve insulin levels and blood pressure. However, early TRF did not affect HDL or LDL cholesterol levels. The most important limitations of this study were the small sample size that completed the trial and the lack of 24 h glucose levels and 24 h blood pressure measurements. Hence, further studies are needed to clearly investigate the best feeding time window [36].

Furthermore, Gabel et al. assessed the potential effects of an 8 h time-restricted feeding regimen versus a no-intervention control group on body weight and metabolic disease risk factors in obese adults [37]. This study did not find any significant differences between groups for diastolic blood pressure, heart rate, total, LDL and HDL cholesterol, triglycerides, glucose, insulin, HOMA-IR, and homocysteine. However, this study suffered from some limitations concerning the lack of a randomized design in the clinical trial, the short duration of the intervention, and the self-reported data, which increased the possibility of recall bias [37]. Accordingly, Martens et al. evaluated the feasibility of 6 weeks of time-restricted vs. normal feeding for improving cardiovascular function in healthy midlife and older adults under free-living conditions [38]. However, the findings did not support any improvement in the primary outcome of vascular endothelial function [38].

Furthermore, Jamshed et al. investigated TRE with eating over a window of 12 or more hours. This study demonstrated that the intervention reduced diastolic blood pressure over a period of 12 or more hours at 14 weeks [39]. However, early TRE did not affect most fasting cardiometabolic risk factors. Thus, it was suggested that future clinical trials should enroll more participants and should carefully examine the eating window [39]. As we mentioned before, Cai et al. also suggested that ADF could be an efficient approach for the effective management of NAFLD with moderate caloric restriction [20].

### 3.4. TRE or TRF and Non-Alcoholic Fatty Liver Disease (NAFLD)

There are certain clinical studies that assessed the potential effect of TRE on non-alcoholic fatty liver disease (NAFLD). The currently existing studies are summarized in Table 4. This table includes five clinical studies with adequate, well-organized methodologies.

Feehan et al. examined whether IF could ameliorate NAFLD progression and improve symptoms’ severity [40]. The TRF group showed significant improvements in hepatic fibrosis predictors like histology. However, the most important drawbacks of this study were the small sample size and the method of NAFLD diagnosis, with ultrasound being the most accurate [40]. In addition, Badran et al. determined the potential short-term effects of Ramadan fasting (RF) as a sort of IF on the biochemical parameters of patients with NAFLD [41]. More to the point, IF significantly reduced liver transaminases, improved lipid profile, and decreased fibrosis score [41]. As we mentioned before, Cai et al. found significant improvements in NAFLD progression [20]. In addition, Ezpeleta et al. conducted a 3-month clinical trial to assess the differences between the intrahepatic triglyceride content of ADF combined with aerobic exercise and ADF alone, exercise alone, and a control group in adults affected by obesity and NAFLD [42]. This study revealed that ADF combined with exercise significantly reduced intrahepatic triglyceride content. However, to the best of our knowledge, there is no other study examining the impact of aerobic exercise on types of diets such as IF, and therefore, future clinical trials should be performed to verify these results over longer durations of time [42]. Accordingly, Wei et al. aimed to compare the potential effects of TRE vs. habitual meal timing on intrahepatic triglyceride content and metabolic risk factors among patients affected by obesity and NAFLD [43]. The 8 h TRE diet was no more effective in reducing the intrahepatic triglyceride content and in achieving resolution of NAFLD among patients with NAFLD than daily CR (habitual meal timing) with the same caloric intake restriction. The main limitation of this study was the use of intrahepatic triglyceride content instead of biopsy. Moreover, since this study began in January 2020, it was impacted by the COVID-19 pandemic [43].

## 4. Discussion

**Figure 3:**
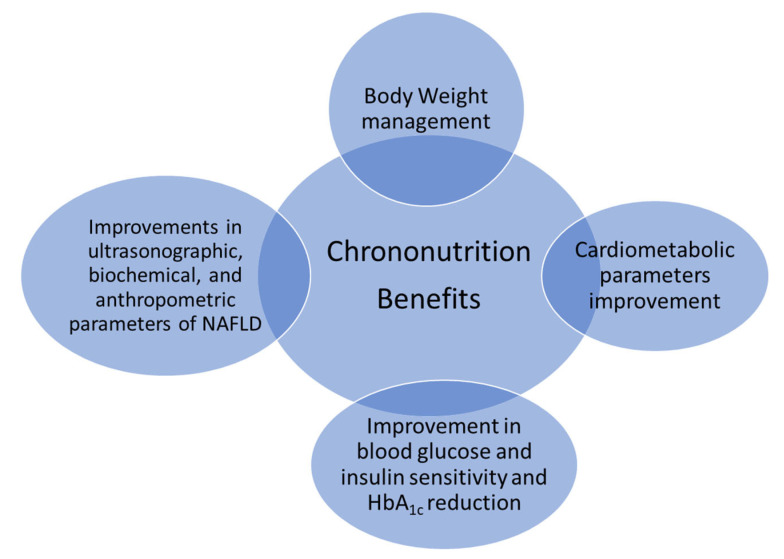
Chrononutrition as a crucial agent for promoting the prevention and effective management of several metabolic disorders.

According to the research literature, chrononutrition investigates the alignment of food intake with circadian rhythms, which exhibits promising benefits for body weight reduction and other cardiovascular risk factors [44]. Thus, based on the existing evidence, chrononutrition could be considered a crucial agent for promoting the prevention and effective management of several metabolic disorders (Figure 3). In this aspect, IF constitutes an umbrella of different diets that involve eating at specific times. Also, it is becoming a promising approach to achieving effective management and prevention of chronic metabolic diseases [45,46]. A growing body of evidence indicates that TRE can produce several metabolic benefits, mainly in body weight and fat loss [47]. To summarize the available evidence, ADF has been associated with a mean weight loss of 0.75 kg per week, whereas the form 5:2 has been associated with a mean weight loss of 0.25 kg per week [48].

In general, the mean duration of the existing studies was approximately 12 weeks with or without simultaneous exercise intervention, which is usually associated with a high degree of diet compliance. In this period, there may be a higher risk of hypoproteinemia/loss of muscle mass; thus, it is important to design and perform future studies [49]. Based on the limited available data, more research will be needed before any solid conclusions can be obtained. The improvements in glucose levels and lipid profile remain controversial since some studies show little or no effect [50]. As it concerns liver diseases, eating patterns, liver homeostasis, circadian clock function and metabolic health are all fundamentally linked to each other. The efficacy of IF was stronger in the management of NAFLD due to body weight and fat loss [51]. Moreover, most of the currently available studies had adequate, well-organized methodologies. The majority of the existing studies have been focused on individuals affected by obesity or diabetes mellitus, while a lower number of studies have been performed on patients with cardiovascular diseases and NAFLD. In addition, favorable effects of IF were observed in religious periods such as Ramadan [41]; however, in specific periods like winter holidays, body weight fluctuations are more commonly observed [52].

The main ways by which IF exerted positive effects on weight loss were reduced energy intake due to the limited eating window, depletion of liver glycogen, and activation of fat storage as energy fuel [53]. Moreover, it can contribute to circadian perturbance and influence the manifestation of metabolic disorders [54]. Indicatively, meal timing and frequency, skipping meals, and fasting are all associated with metabolic syndrome. Eating frequent meals and eating in the morning could exert a protective effect on metabolic syndrome. On the other hand, eating at night, skipping breakfast, eating one meal per day, and eating irregularly could facilitate the development of metabolic syndrome risks in adults [54]. Apart from body weight loss, in the bibliography, there were several cellular mechanisms that suggested positive effects on glucose regulation [53,55]. Notably, incretin hormones, especially glucagon-like peptide-1 (GLP-1) and glucose-dependent insulinotropic polypeptide (GIP), show diurnal variation and are augmented in the early part of the day, leading to a more rapid insulin response to nutrient intake in the morning. Comparing isocaloric meals of identical macronutrient composition consumed at 08.00 h and 17.00 h, the morning meal led to rapid increases in both GLP-1 and GIP, leading to rapid insulin response and lower post-prandial total and peak glucose levels [56]. These variations in morning metabolic physiology could contribute to the ‘second meal phenomenon’, which is depicted by an attenuated increase in blood glucose levels in response to a second meal when preceded by a prior meal earlier in the day [55,56]. However, patients with type 2 diabetes who take both insulin and oral hypoglycemic medication may be at higher risk of developing hypoglycemia; thus, it is important to design and perform future studies to clarify this issue.

Analyzing the concept of chrononutrition, the recent COVID-19 lockdown and its confinement measures should also be taken into consideration, as the COVID-19 pandemic has exerted a significant impact on people’s daily lives worldwide. In this aspect, an emerging body of research suggests that personality traits capture the individual differences observed in health behaviors during the COVID-19 pandemic [57]. A recent exploratory study has highlighted the substantial impact of the COVID-19 lockdown on the association between personality and chrononutrition, emphasizing the significance of addressing differences in how individuals respond to the pandemic and understanding how personality can predict what and when people eat when faced with a novel environment [58]. Notably, during the first COVID-19 lockdown in Italy, a cross-sectional study revealed a change in eating habits for 58% of its participants in terms of mealtimes or content of meals. Being an evening chronotype and experiencing poor sleep implied a higher risk of changing eating habits, including a delay in the timing of meals [59]. A nested case–control study also showed that during the COVID-19 pandemic, its participants consumed their breakfast less regularly than before the COVID-19 pandemic, suggesting that meal regularity declined during the COVID-19 pandemic, while meal frequency, especially snack consumption, increased [60].

There is also recent evidence that chrononutrition behaviors such as TRE or TRF may have a potential impact on emotional state and mood. In this aspect, Murta et al., have suggested that IF may exert positive effects for the treatment of mood disorders [61]. However, most of the available clinical trials have specific limitations, such as small sample sizes and uncontrolled designs, highlighting the need to perform better-designed studies and controlled evaluations to assess its efficiency in the treatment of major depression [61].

Collectively, there are some limitations of the studies reviewed. In fact, most of the studies had a small sample size and a short duration of intervention. Thus, it is important to design and perform future studies with longer duration and larger population-based sample sizes [62,63]. Moreover, it is important to determine the best timing of the eating window and its feasibility [8]. In addition, it is very important to clarify the individuals’ chronotype when choosing the most efficient meal timing [64]. The ideal duration of such types of nutritional intervention, such as TRE or TRF, should also be specified, after which a less time-restricted diet could be applied for maintaining body weight loss and the other beneficial effects of TRE or TRF throughout a lifetime [65]. Additionally, future studies should take into consideration diverse potential confounding factors that may affect their final outcomes, such as sociodemographic (age, sex, nationality, education, economic status, and family/marital status) and lifestyle (e.g., physical and mental health) factors.

## 5. Conclusions

In the last few years, there has been a gradually increasing number of clinical studies evaluating the potential beneficial effects of specific chrononutrition behaviors, such as TRE or TRF. These studies focus on the prevention or co-treatment of diverse chronic diseases, with a particular emphasis on metabolic disorders. They especially examine the reduction of body weight, body composition, blood glucose, insulin, and triglycerides in individuals with excessive body weight or body weight-related metabolic disorders. Several clinical studies revealed promising beneficial effects of the chrononutrition concept on metabolic disorders, promoting metabolic health. However, several of them have serious limitations. In this aspect, future well-designed clinical studies with an adequate nutritional intervention duration are highly recommended in order for more precise conclusions to be drawn. The long-term effects of the COVID-19 pandemic and their potential impact on chrononutrition behaviors should also be taken into account in future studies.

## Figures and Tables

**Table 1:** Clinical studies assessing the effect of TRE or TRF on obesity.

Study Type	Author (Country)	Study Population	Basic Results	References
Randomized controlled trial	Catenacci et al. (USA)	N = 26, obese female and male (ADF group N = 34, 16/8 TRF group N = 33, control group N = 34)	No significant difference in weight loss between groups.	[18]
Randomized controlled trial	Chair et al. (China)	N = 101, overweight or obese female and male	The reductions in body weight, body mass index, and waist circumference in the ADF and 16/8 TRF groups were more significant than those in the control group across the study period.	[19]
Randomized controlled trial	Cai et al. (China)	N = 271, female and male with NAFLD (ADF group, TRF group)	Both groups decreased body weight after 12 weeks.	[20]
Single-arm, paired-sample clinical trial	Wilkinson et al. (USA)	N = 19, overweight or obese female and male	Body weight was decreased after TRE intervention.	[21]
Prospective study	Gabel et al. (USA)	N = 14, obese female and male	Body weight was decreased after TRE intervention.	[22]
Randomized controlled trial	Schübel et al. (Germany)	N = 150, overweight or obese female and male	ICR and CCR are effective methods for weight loss.	[23]
Randomized controlled trial	Sundfør et al. (Norway)	N = 112, overweight or obese female and male	Both intermittent and continuous energy restriction resulted in similar weight loss.	[24]
Randomized clinical trial	Lowe et al. (USA)	N = 116, overweight or obese female and male	There was a significant decrease in weight in the TRE but no significant change in the consistent meal timing (CMT) group.	[25]
Non-randomized clinical trial	Schroder et al. (Brazil)	N = 32, obese women	TRF reduced body weight (~4 kg).	[26]

**Table 2:** Clinical studies assessing the effect of TRE or TRF in diabetes mellitus.

Study Type	Author (Country)	Study Population	Basic Results	References
Randomized controlled trial	Che et al. (China)	N = 104, overweight female and male with type 2 diabetes	Improvement in blood glucose and insulin sensitivity, quality of life, and reduction in the dosage of hypoglycemic drugs.	[27]
Randomized clinical trial	Pavlou et al. (USA)	N = 69, overweight female and male with type 2 diabetes	Greater weight loss and no differences in hemoglobin A_1c_ (HbA_1c_); no difference between the TRE and CR groups compared with controls.	[28]
Single-arm, paired-sample trial	Wilkinson et al. (USA)	N = 19, overweight or obese female and male with metabolic syndrome	Reduction in HbA_1c_.	[21]
Randomized controlled trial	Chair et al. (China)	N = 101, overweight or obese female and male with prediabetes (ADF group N = 34, 16/8 TRF group N = 33, control group N = 34)	Significant reductions in blood glucose were observed.	[19]
Randomized controlled trial	Carter et al. (Australia)	N = 137, female and male with type 2 diabetes	No significant differences between groups in body composition, fasting glucose levels, lipid levels, or total medication effect score at 24 months.	[29]
Randomized controlled trial	Gabel et al. (USA)	N = 43, overweight or obese female and male with insulin resistance	Improvements in HOMA-IR due to ADF vs. daily CR.	[30]
Randomized controlled trial	Kunduraci et al. (Turkey)	N = 65, female and male with metabolic syndrome	No significant differences were observed in fasting plasma glucose, insulin, HbA1c, HOMA-IR in both groups.	[31]
Randomized controlled trial	Obermayer et al. (Austria)	Ν = 47, female and male with diabetes mellitus	The IF group showed a significant HbA1c reduction.	[32]
Controlled clinical trial	Peeke et al. (USA)	Ν = 60, obese female and male	The differences between groups were not significantly different.	[33]
Clinical trial	Khalfallah et al. (Egypt)	N = 485, prediabetic female and male	Combined with LCD, was associated with lower progression to diabetes mellitus and lower incidence of implications.	[34]

**Table 3:** Clinical studies assessing the effect of TRE or TRF on cardiovascular diseases.

Study Type	Author (Country)	Study Population	Basic Results	References
Randomized controlled trial	Cienfuegos et al. (USA)	N = 59, obese female and male	Neither intervention had any effect on plasma lipid levels.	[35]
Randomized controlled trial	Sutton et al. (USA)	N = 8 men with prediabetes	Early time-restricted feeding (eTRF) improved insulin sensitivity, β cell responsiveness, blood pressure, oxidative stress, and appetite.	[36]
Clinical trial	Chow et al. (USA)	N = 46 obese female and male	Diastolic blood pressure, LDL cholesterol, HDL cholesterol, triglycerides, and homocysteine were not significantly different from controls after 12 weeks.	[37]
Randomized controlled trial	Martens et al. (USA)	N = 22, non-obese, healthy female and male	No significant differences in cardiometabolic risk factors.	[38]
Randomized controlled trial	Jamshed et al. (USA)	N = 90, obese female and male	Lower diastolic blood pressure and no differences in systolic blood pressure, heart rate or plasma lipid levels.	[39]
Randomized controlled trial	Cai et al. (China)	N = 271, female and male with NAFLD (ADF group vs. TRF group)	Intermittent fasting led to improvements in NAFLD due to fat loss.	[20]

**Table 4:** Clinical studies assessing the effect of TRE or TRF on NAFLD.

Study Type	Author (Country)	Study Population	Basic Results	References
Single-blind randomized controlled trial	Feehan et al. (Australia)	N = 28, female and male with NAFLD	Intermittent fasting significantly decreased hepatic steatosis.	[40]
Clinical trial	Badran et al. (Egypt)	N = 98, NAFLD female and male	Intermittent fasting led to improvements in ultrasonographic, biochemical, and anthropometric parameters of NAFLD.	[41]
Randomized controlled trial	Cai et al. (China)	N = 271, female and male with NAFLD (ADF group vs. TRF group)	Intermittent fasting led to improvements in NAFLD due to fat loss.	[20]
Randomized controlled trial	Ezpeleta et al. (USA)	N = 80, obese female and male with NAFLD	Intrahepatic triglyceride (IHTG) content reduced in combination group (ADF + exercise).	[42]
Randomized controlled trial	Wei et al. (China)	N = 88, obese female and male with NAFLD	TRE did not produce additional benefits for reducing IHTG content, body fat, and metabolic risk factors compared with habitual meal timing.	[43]

## Data Availability

The data of the present study are available upon request from the corresponding author.

## References

[1] Codoñer-Franch P., Gombert M., Martínez-Raga J., Cenit M.C. (2023). Circadian Disruption and Mental Health: The Chronotherapeutic Potential of Microbiome-Based and Dietary Strategies. Int. J. Mol. Sci..

[2] Ahluwalia M.K. (2022). Chrononutrition-When We Eat Is of the Essence in Tackling Obesity. Nutrients.

[3] Chaix A., Manoogian E.N.C., Melkani G.C., Panda S. (2019). Time-Restricted Eating to Prevent and Manage Chronic Metabolic Diseases. Annu. Rev. Nutr..

[4] Perez-Diaz-Del-Campo N., Castelnuovo G., Caviglia G.P., Armandi A., Rosso C., Bugianesi E. (2022). Role of Circadian Clock on the Pathogenesis and Lifestyle Management in Non-Alcoholic Fatty Liver Disease. Nutrients.

[5] Lavallee C.M., Bruno A., Ma C., Raman M. (2022). The Role of Intermittent Fasting in the Management of Nonalcoholic Fatty Liver Disease: A Narrative Review. Nutrients.

[6] Tippairote T., Janssen S., Chunhabundit R. (2021). Restoration of metabolic tempo through time-restricted eating (TRE) as the preventive measure for metabolic diseases. Crit. Rev. Food Sci. Nutr..

[7] Moon S., Kang J., Kim S.H., Chung H.S., Kim Y.J., Yu J.M., Cho S.T., Oh C.M., Kim T. (2020). Beneficial Effects of Time-Restricted Eating on Metabolic Diseases: A Systemic Review and Meta-Analysis. Nutrients.

[8] Kamarul Zaman M., Teng N., Kasim S.S., Juliana N., Alshawsh M.A. (2023). Effects of time-restricted eating with different eating duration on anthropometrics and cardiometabolic health: A systematic review and meta-analysis. World J. Cardiol..

[9] Mishra S., Persons P.A., Lorenzo A.M., Chaliki S.S., Bersoux S. (2023). Time-Restricted Eating and Its Metabolic Benefits. J. Clin. Med..

[10] Lange M., Nadkarni D., Martin L., Newberry C., Kumar S., Kushner T. (2023). Intermittent fasting improves hepatic end points in nonalcoholic fatty liver disease: A systematic review and meta-analysis. Hepatol. Commun..

[11] Manoogian E.N.C., Chow L.S., Taub P.R., Laferrère B., Panda S. (2022). Time-restricted Eating for the Prevention and Management of Metabolic Diseases. Endocr. Rev..

[12] Schuppelius B., Peters B., Ottawa A., Pivovarova-Ramich O. (2021). Time Restricted Eating: A Dietary Strategy to Prevent and Treat Metabolic Disturbances. Front. Endocrinol..

[13] Kirkham A.A., Parr E.B., Kleckner A.S. (2022). Cardiometabolic health impacts of time-restricted eating: Implications for type 2 diabetes, cancer and cardiovascular diseases. Curr. Opin. Clin. Nutr. Metab. Care.

[14] Sun J.C., Tan Z.T., He C.J., Hu H.L., Zhai C.L., Qian G. (2023). Time-restricted eating with calorie restriction on weight loss and cardiometabolic risk: A systematic review and meta-analysis. Eur. J. Clin. Nutr..

[15] Silva A.I., Direito M., Pinto-Ribeiro F., Ludovico P., Sampaio-Marques B. (2023). Effects of Intermittent Fasting on Regulation of Metabolic Homeostasis: A Systematic Review and Meta-Analysis in Health and Metabolic-Related Disorders. J. Clin. Med..

[16] Nowosad K., Sujka M. (2021). Effect of Various Types of Intermittent Fasting (IF) on Weight Loss and Improvement of Diabetic Parameters in Human. Curr. Nutr. Rep..

[17] Naous E., Achkar A., Mitri J. (2023). Intermittent Fasting and Its Effects on Weight, Glycemia, Lipids, and Blood Pressure: A Narrative Review. Nutrients.

[18] Catenacci V.A., Pan Z., Ostendorf D., Brannon S., Gozansky W.S., Mattson M.P., Martin B., MacLean P.S., Melanson E.L., Troy Donahoo W. (2016). A randomized pilot study comparing zero-calorie alternate-day fasting to daily caloric restriction in adults with obesity. Obesity.

[19] Chair S.Y., Cai H., Cao X., Qin Y., Cheng H.Y., Ng M.T. (2022). Intermittent Fasting in Weight Loss and Cardiometabolic Risk Reduction: A Randomized Controlled Trial. J. Nurs. Res..

[20] Cai H., Qin Y.L., Shi Z.Y., Chen J.H., Zeng M.J., Zhou W., Chen R.Q., Chen Z.Y. (2019). Effects of alternate-day fasting on body weight and dyslipidaemia in patients with non-alcoholic fatty liver disease: A randomised controlled trial. BMC Gastroenterol..

[21] Wilkinson M.J., Manoogian E.N.C., Zadourian A., Lo H., Fakhouri S., Shoghi A., Wang X., Fleischer J.G., Navlakha S., Panda S. (2020). Ten-Hour Time-Restricted Eating Reduces Weight, Blood Pressure, and Atherogenic Lipids in Patients with Metabolic Syndrome. Cell Metab..

[22] Gabel K., Marcell J., Cares K., Kalam F., Cienfuegos S., Ezpeleta M., Varady K.A. (2020). Effect of time restricted feeding on the gut microbiome in adults with obesity: A pilot study. Nutr. Health.

[23] Schübel R., Nattenmüller J., Sookthai D., Nonnenmacher T., Graf M.E., Riedl L., Schlett C.L., von Stackelberg O., Johnson T., Nabers D. (2018). Effects of intermittent and continuous calorie restriction on body weight and metabolism over 50 wk: A randomized controlled trial. Am. J. Clin. Nutr..

[24] Sundfør T.M., Svendsen M., Tonstad S. (2018). Effect of intermittent versus continuous energy restriction on weight loss, maintenance and cardiometabolic risk: A randomized 1-year trial. Nutr. Metab. Cardiovasc. Dis..

[25] Lowe D.A., Wu N., Rohdin-Bibby L., Moore A.H., Kelly N., Liu Y.E., Philip E., Vittinghoff E., Heymsfield S.B., Olgin J.E. (2020). Effects of Time-Restricted Eating on Weight Loss and Other Metabolic Parameters in Women and Men With Overweight and Obesity: The TREAT Randomized Clinical Trial. JAMA Intern. Med..

[26] Schroder J.D., Falqueto H., Mânica A., Zanini D., de Oliveira T., de Sá C.A., Cardoso A.M., Manfredi L.H. (2021). Effects of time-restricted feeding in weight loss, metabolic syndrome and cardiovascular risk in obese women. J. Transl. Med..

[27] Che T., Yan C., Tian D., Zhang X., Liu X., Wu Z. (2021). Time-restricted feeding improves blood glucose and insulin sensitivity in overweight patients with type 2 diabetes: A randomised controlled trial. Nutr. Metab..

[28] Pavlou V., Cienfuegos S., Lin S., Ezpeleta M., Ready K., Corapi S., Wu J., Lopez J., Gabel K., Tussing-Humphreys L. (2023). Effect of Time-Restricted Eating on Weight Loss in Adults with Type 2 Diabetes: A Randomized Clinical Trial. JAMA Netw. Open.

[29] Carter S., Clifton P.M., Keogh J.B. (2019). The effect of intermittent compared with continuous energy restriction on glycaemic control in patients with type 2 diabetes: 24-month follow-up of a randomised noninferiority trial. Diabetes Res. Clin. Pract..

[30] Gabel K., Kroeger C.M., Trepanowski J.F., Hoddy K.K., Cienfuegos S., Kalam F., Varady K.A. (2019). Differential Effects of Alternate-Day Fasting Versus Daily Calorie Restriction on Insulin Resistance. Obesity.

[31] Kunduraci Y.E., Ozbek H. (2020). Does the Energy Restriction Intermittent Fasting Diet Alleviate Metabolic Syndrome Biomarkers? A Randomized Controlled Trial. Nutrients.

[32] Obermayer A., Tripolt N.J., Pferschy P.N., Kojzar H., Aziz F., Müller A., Schauer M., Oulhaj A., Aberer F., Sourij C. (2023). Efficacy and Safety of Intermittent Fasting in People With Insulin-Treated Type 2 Diabetes (INTERFAST-2)-A Randomized Controlled Trial. Diabetes Care.

[33] Peeke P.M., Greenway F.L., Billes S.K., Zhang D., Fujioka K. (2021). Effect of time restricted eating on body weight and fasting glucose in participants with obesity: Results of a randomized, controlled, virtual clinical trial. Nutr. Diabetes.

[34] Khalfallah M., Elnagar B., Soliman S.S., Eissa A., Allaithy A. (2023). The Value of Intermittent Fasting and Low Carbohydrate Diet in Prediabetic Patients for the Prevention of Cardiovascular Diseases. Arq. Bras. Cardiol..

[35] Cienfuegos S., Gabel K., Kalam F., Ezpeleta M., Wiseman E., Pavlou V., Lin S., Oliveira M.L., Varady K.A. (2020). Effects of 4- and 6-h Time-Restricted Feeding on Weight and Cardiometabolic Health: A Randomized Controlled Trial in Adults with Obesity. Cell Metab..

[36] Sutton E.F., Beyl R., Early K.S., Cefalu W.T., Ravussin E., Peterson C.M. (2018). Early Time-Restricted Feeding Improves Insulin Sensitivity, Blood Pressure, and Oxidative Stress Even without Weight Loss in Men with Prediabetes. Cell Metab..

[37] Chow L.S., Manoogian E.N.C., Alvear A., Fleischer J.G., Thor H., Dietsche K., Wang Q., Hodges J.S., Esch N., Malaeb S. (2020). Time-Restricted Eating Effects on Body Composition and Metabolic Measures in Humans who are Overweight: A Feasibility Study. Obesity.

[38] Martens C.R., Rossman M.J., Mazzo M.R., Jankowski L.R., Nagy E.E., Denman B.A., Richey J.J., Johnson S.A., Ziemba B.P., Wang Y. (2020). Short-term time-restricted feeding is safe and feasible in non-obese healthy midlife and older adults. Geroscience.

[39] Jamshed H., Steger F.L., Bryan D.R., Richman J.S., Warriner A.H., Hanick C.J., Martin C.K., Salvy S.J., Peterson C.M. (2022). Effectiveness of Early Time-Restricted Eating for Weight Loss, Fat Loss, and Cardiometabolic Health in Adults with Obesity: A Randomized Clinical Trial. JAMA Intern. Med..

[40] Feehan J., Mack A., Tuck C., Tchongue J., Holt D.Q., Sievert W., Moore G.T., de Courten B., Hodge A. (2023). Time-Restricted Fasting Improves Liver Steatosis in Non-Alcoholic Fatty Liver Disease-A Single Blinded Crossover Trial. Nutrients.

[41] Badran H., Elsabaawy M., Sakr A., Eltahawy M., Elsayed M., Elsabaawy D.M., Abdelkreem M. (2022). Impact of intermittent fasting on laboratory, radiological, and anthropometric parameters in NAFLD patients. Clin. Exp. Hepatol..

[42] Ezpeleta M., Gabel K., Cienfuegos S., Kalam F., Lin S., Pavlou V., Song Z., Haus J.M., Koppe S., Alexandria S.J. (2023). Effect of alternate day fasting combined with aerobic exercise on non-alcoholic fatty liver disease: A randomized controlled trial. Cell Metab..

[43] Wei X., Lin B., Huang Y., Yang S., Huang C., Shi L., Liu D., Zhang P., Lin J., Xu B. (2023). Effects of Time-Restricted Eating on Nonalcoholic Fatty Liver Disease: The TREATY-FLD Randomized Clinical Trial. JAMA Netw. Open.

[44] Katsi V., Papakonstantinou I.P., Soulaidopoulos S., Katsiki N., Tsioufis K. (2022). Chrononutrition in Cardiometabolic Health. J. Clin. Med..

[45] Gabel K., Varady K.A. (2022). Current research: Effect of time restricted eating on weight and cardiometabolic health. J. Physiol..

[46] Kazeminasab F., Baharlooie M., Karimi B., Mokhtari K., Rosenkranz S.K., Santos H.O. (2023). Effects of intermittent fasting combined with physical exercise on cardiometabolic outcomes: Systematic review and meta-analysis of clinical studies. Nutr. Rev..

[47] Ezpeleta M., Cienfuegos S., Lin S., Pavlou V., Gabel K., Tussing-Humphreys L., Varady K.A. (2023). Time-restricted eating: Watching the clock to treat obesity. Cell Metab..

[48] Katsarou A.L., Katsilambros N.L., Koliaki C.C. (2021). Intermittent Energy Restriction, Weight Loss and Cardiometabolic Risk: A Critical Appraisal of Evidence in Humans. Healthcare.

[49] Schroor M.M., Joris P.J., Plat J., Mensink R.P. (2024). Effects of Intermittent Energy Restriction Compared with Those of Continuous Energy Restriction on Body Composition and Cardiometabolic Risk Markers—A Systematic Review and Meta-Analysis of Randomized Controlled Trials in Adults. Adv. Nutr..

[50] Van den Burg E.L., van Peet P.G., Schoonakker M.P., van de Haar D.E., Numans M.E., Pijl H. (2023). Metabolic impact of intermittent energy restriction and periodic fasting in patients with type 2 diabetes: A systematic review. Nutr. Rev..

[51] Marjot T., Tomlinson J.W., Hodson L., Ray D.W. (2023). Timing of energy intake and the therapeutic potential of intermittent fasting and time-restricted eating in NAFLD. Gut.

[52] Abdulan I.M., Popescu G., Maștaleru A., Oancea A., Costache A.D., Cojocaru D.-C., Cumpăt C.-M., Ciuntu B.M., Rusu B., Leon M.M. (2023). Winter Holidays and Their Impact on Eating Behavior—A Systematic Review. Nutrients.

[53] Tsitsou S., Zacharodimos N., Poulia K.A., Karatzi K., Dimitriadis G., Papakonstantinou E. (2022). Effects of Time-Restricted Feeding and Ramadan Fasting on Body Weight, Body Composition, Glucose Responses, and Insulin Resistance: A Systematic Review of Randomized Controlled Trials. Nutrients.

[54] Alkhulaifi F., Darkoh C. (2022). Meal Timing, Meal Frequency and Metabolic Syndrome. Nutrients.

[55] Flanagan A., Bechtold D.A., Pot G.K., Johnston J.D. (2021). Chrono-nutrition: From molecular and neuronal mechanisms to human epidemiology and timed feeding patterns. J. Neurochem..

[56] Lindgren O., Mari A., Deacon C.F., Carr R.D., Winzell M.S., Vikman J., Ahrén B. (2009). Differential islet and incretin hormone responses in morning versus afternoon after standardized meal in healthy men. J. Clin. Endocrinol. Metab..

[57] Kekäläinen T., Hietavala E.M., Hakamäki M., Sipilä S., Laakkonen E.K., Kokko K. (2021). Personality Traits and Changes in Health Behaviors and Depressive Symptoms during the COVID-19 Pandemic: A Longitudinal Analysis from Pre-pandemic to Onset and End of the Initial Emergency Conditions in Finland. Int. J. Environ. Res. Public Health.

[58] Al-Abdi T., Heraclides A., Papageorgiou A., Philippou E. (2022). The Effect of Personality on Chrononutrition during the COVID-19 Lockdown in Qatar. Nutrients.

[59] Bazzani A., Marantonio S., Andreozzi G., Lorenzoni V., Bruno S., Cruz-Sanabria F., d′Ascanio P., Turchetti G., Faraguna U. (2022). Late chronotypes, late mealtimes. Chrononutrition and sleep habits during the COVID-19 lockdown in Italy. Appetite.

[60] Saals B., Boss H.M., Pot G.K. (2022). Young people and adolescents have more irregular meals during the COVID-19 pandemic: A nested case-control study on chrono-nutrition before and during the COVID-19 pandemic. Chronobiol. Int..

[61] Murta L., Seixas D., Harada L., Damiano R.F., Zanetti M. (2023). Intermittent Fasting as a Potential Therapeutic Instrument for Major Depression Disorder: A Systematic Review of Clinical and Preclinical Studies. Int. J. Mol. Sci..

[62] Soliman G.A. (2022). Intermittent fasting and time-restricted eating role in dietary interventions and precision nutrition. Front. Public Health.

[63] Chavanne A., Jacobi D. (2024). Precision medicine in endocrinology: Unraveling metabolic health through time-restricted eating. Ann. Endocrinol..

[64] Mazri F.H., Manaf Z.A., Shahar S., Mat Ludin A.F. (2019). The Association between Chronotype and Dietary Pattern among Adults: A Scoping Review. Int. J. Environ. Res. Public Health.

[65] Munhoz A.C., Vilas-Boas E.A., Panveloski-Costa A.C., Leite J.S.M., Lucena C.F., Riva P., Emilio H., Carpinelli A.R. (2020). Intermittent Fasting for Twelve Weeks Leads to Increases in Fat Mass and Hyperinsulinemia in Young Female Wistar Rats. Nutrients.

